# Royal Medical and Chirurgical Society

**Published:** 1897-01-30

**Authors:** 


### Medical Progress and Hospital Clinics.

[*The Editor will be glad to receive offers of co-operation and contributions from members of the profession. All letters should be addressed to* The Editor, at the Office, 28 & 29, Southampton Street, Strand, London, W.C.]

### ROYAL MEDICAL AND CHIRURGICAL SOCIETY.

There are few emergencies more urgent than the occurrence of perforation of the intestine in the course of typhoid fever. Such perforation is so fatal an accident, and the success which has of late attended the operation of laparotomy and closure of the opening in cases of perforating ulcers of the stomach has been so considerable, that one need not wonder that, on the one hand, physicians have been ready to appeal to surgeons for assistance in such cases, nor, on the other, that surgeons should be beginning to look on cases of perforated intestines as legitimately coming within their province, even though they occur in the midst of so serious a condition as typhoid fever. Hitherto, however, the success which has attended surgical interference in this field has not been great, and, in addition to the want of success, the difficulty of diagnosis—the almost impossibility of determining with absolute certainty whether or no the symptoms in a given case are due to perforation or not—has tended to prevent a more frequent resort to surgical measures in the treatment of this most serious accident.

In the papers read at the meeting of the Royal Medical and Chirurgical Society on January 26th all these points were illustrated. In Dr. Lauder Brunton's case, in which perforation was found and treated, the symptoms were less marked and far less characteristic of perforation than they were in that related by Dr. Herringham, in which, on laparotomy being performed, no lesion of the intestine was found. In both these cases the operation was performed by Mr. Bowlby, and in both recovery took place.

A case related by Dr. Francis Hawkins, in which after death from typhoid fever an ulcer was found in the gall bladder, led Mr. Marmaduke Sheild to mention a case in which he had operated for what was imagined to be perforation of the intestine during typhoid fever, and in which it was only after twenty minutes' careful search that the seat of perforation was found to be in the gall bladder, where there was what seemed like a typical typhoid ulcer. This was closed, and the patient recovered. The case, however, served to illustrate the difficulty which lay in the way of certain diagnosis in cases of perforation of the intestine.

Dr. Brunton's patient was a man, aged thirty-seven, who was admitted into St. Bartholomew's Hospital on October 3rd, 1895, at about the end of the second week of typhoid fever. The case was a severe one; there was hæmorrhage soon after admission, a relapse began on October 30th which lasted nearly a fortnight, and convalescence was slow. On December 14th there was a sudden attack of severe abdominal pain, followed after some hours by abdominal distension and symptoms of obstruction, but without nausea or vomiting. Laparotomy was performed about eighteen hours from the commencement of the pain, the incision being made in the middle line below the umbilicus. The upper part of the peritoneal cavity was free from gas or fæcal matter, but the great omentum was adherent in the pelvis, and under it was found a very indurated coil of bowel, which had been perforated by a small ulcer about as large as a pea, situated opposite to the attachment of the mesentery. This ulcer was closed by ten silk sutures after Lembert's method transversely to the long axis of the bowel. The abdomen was irrigated with hot water, a drainage-tube passed into the pelvis, and the wound sutured.

Recovery was uninterrupted, the temperature remaining normal throughout. The tube was removed in forty-eight hours, and for four days no food was given by the mouth, except water in teaspoonful doses. The bowels acted on the fifth day, after the use of an enema.

It was pointed out that the case was one unusually well adapted for operative treatment, and could not be compared with those cases where perforation occurred during the height of the fever and in a patient greatly prostrated by the disease. It was probable that the opening in the bowel had really existed for some time, and had been temporarily but insecurely closed by adhesions. These had gradually yielded when a fuller diet was allowed, and had permitted an escape of the contents of the intestine.

Dr. Herringham's patient was a girl thirteen years of age, who had been in hospital a fortnight suffering from the later stages of a comparatively mild attack of typhoid fever, when, on what was probably about the twenty-eighth day of the disease, she was suddenly attacked with acute abdominal pain, which caused her to cry out constantly. This gradually became worse. She vomited frequently. Her pulse was 140, and small. Morphia gave only temporary relief, and an oil enema had no effect. The abdomen was full and tense; its walls were rigid, and scarcely moved with respiration; the liver dulness was natural; there was marked tenderness on palpation, especially near the umbilicus, to which region the pain was referred. The temperature had remained stationary. There was a general aspect of considerable collapse. A few hours later the child lay with the thighs drawn up, and screamed with pain on the slightest movement; retching was almost constant, although but little was now brought up; the pulse was rapid; and the hands and feet were cold and clammy. On laparotomy being performed, however, no perforation was found nor any evidence of peritonitis.

In the discussion Dr. Lauder Brunton related a case which he had seen quite recently in which symptoms of perforation came on twelve days after admission in a patient who had been suffering from symptoms of typhoid fever for three weeks before he came into hospital. The patient was in very bad condition, having suffered from sloughing and from hæmorrhage, and in view of the apparent hopelessness of the case Dr. Brunton had hesitated to recommend an operation. At the post-mortem examination, however, it was found that the opening was very small, only the size of a pin's head in fact, and so placed that it seemed within the reach of surgical intervention. Truly, all the large intestine was deeply ulcerated as far as the sigmoid flexure; nevertheless he could not but question whether an operation might not have given the man a chance, one which he did not possess while the perforation and extravasation were left untreated; and he hoped that with more experience the operation might be attended with more success than hitherto.

